# Combined Treatment of Hyperbaric Oxygen and Anti-vascular Endothelial Growth Factor Therapy in Cilioretinal Artery Occlusion Associated With Central Retinal Vein Occlusion

**DOI:** 10.7759/cureus.96735

**Published:** 2025-11-13

**Authors:** Júlio Brissos, Rita Pinto, Sara Frazão, Vanda Nogueira

**Affiliations:** 1 Ophthalmology, Unidade Local de Saúde de São José, Lisbon, PRT; 2 Ophthalmology, ALM Primum Oftalmologia, Lisbon, PRT

**Keywords:** anti-vegf therapy, central retinal vein occlusion (crvo), cilioretinal artery occlusion, heterozygous factor v leiden mutation, hyperbaric oxygen therapy, retinal venous pressure

## Abstract

We report a case of a male in his early 40s who presented with transient episodes of visual blurring and was initially diagnosed with isolated cilioretinal artery occlusion (CLRAO). Hyperbaric oxygen therapy (HOT) was initiated on the same day as the onset of the preceding episode, and six sessions had been completed when he was first examined at our department. Despite ongoing HOT, he developed worsening signs of venous stasis. Further investigation confirmed CLRAO secondary to central retinal vein occlusion (CRVO) and revealed heterozygosity for the factor V Leiden mutation. HOT was continued for two weeks, and a single intravitreal injection of bevacizumab (anti-vascular endothelial growth factor (anti-VEGF)) was administered, resulting in full and sustained anatomical and functional recovery, with visual acuity improving from 8/10 to 10/10. This case supports the benefit of anti-VEGF therapy in combined CLRAO and CRVO without exudative or neovascular complications, by promoting vasoconstriction and reducing venous permeability. It also suggests a synergistic effect with HOT by further lowering retinal venous pressure through distinct mechanisms. These findings highlight the potential benefit of addressing both retinal hypoxia and venous hypertension through the combined use of HOT and anti-VEGF therapy in selected cases of mixed retinal vascular occlusion.

## Introduction

Cilioretinal artery occlusion (CLRAO) is a rare event and may present as an isolated finding or as an event secondary to central retinal vein occlusion (CRVO) or to giant cell arteritis [1]. The most common form is the association with CRVO, where increased retinal venous pressure (RVP) leads to CLRAO due to a lack of an autoregulatory mechanism of the cilioretinal artery [2,3]. In this setting, venous stasis and elevated RVP may impede blood flow through the cilioretinal artery, resulting in reversible or permanent occlusion, depending on how severe the retinal venous stasis is and how rapidly collateral circulation is established [3,4]. This interplay creates diagnostic challenges, as the hemodynamic dysfunction might be transient, lasting from a few hours to several days. Furthermore, when a CRVO is fully established, a small CLRAO may be overlooked due to the presence of retinal hemorrhages and ischemia [1,3]. Early recognition is therefore clinically important: if venous stasis persists, reversible hypoperfusion can progress to permanent inner retinal infarction with a fixed scotoma and loss of visual acuity [2-4].

To date, no treatment has been proven effective for CLRAO associated with CRVO. However, some reports have suggested that hyperbaric oxygen therapy (HOT) may be beneficial by enhancing oxygen diffusion to the inner retina and RVP [5]. Intravitreal anti-vascular endothelial growth factor (anti-VEGF) injections have also been used in five published similar cases [3,6,7]. We present a case of CLRAO associated with CRVO treated with a combination of HOT and intravitreal anti-VEGF and discuss the potential synergistic mechanisms underlying this therapeutic approach.

## Case presentation

A male in his early 40s with no known medical history presented to our emergency department with a band-shaped scotoma in the left eye, which he noticed upon waking. He had recently been hospitalized at another institution with a diagnosis of isolated CLRAO, following two transient episodes of the same symptoms occurring over the last three months. A thorough work-up for causes of arterial occlusion had reportedly shown no abnormalities. He had been started on a course of HOT, which was ongoing when we first saw him.

**Figure 1:**
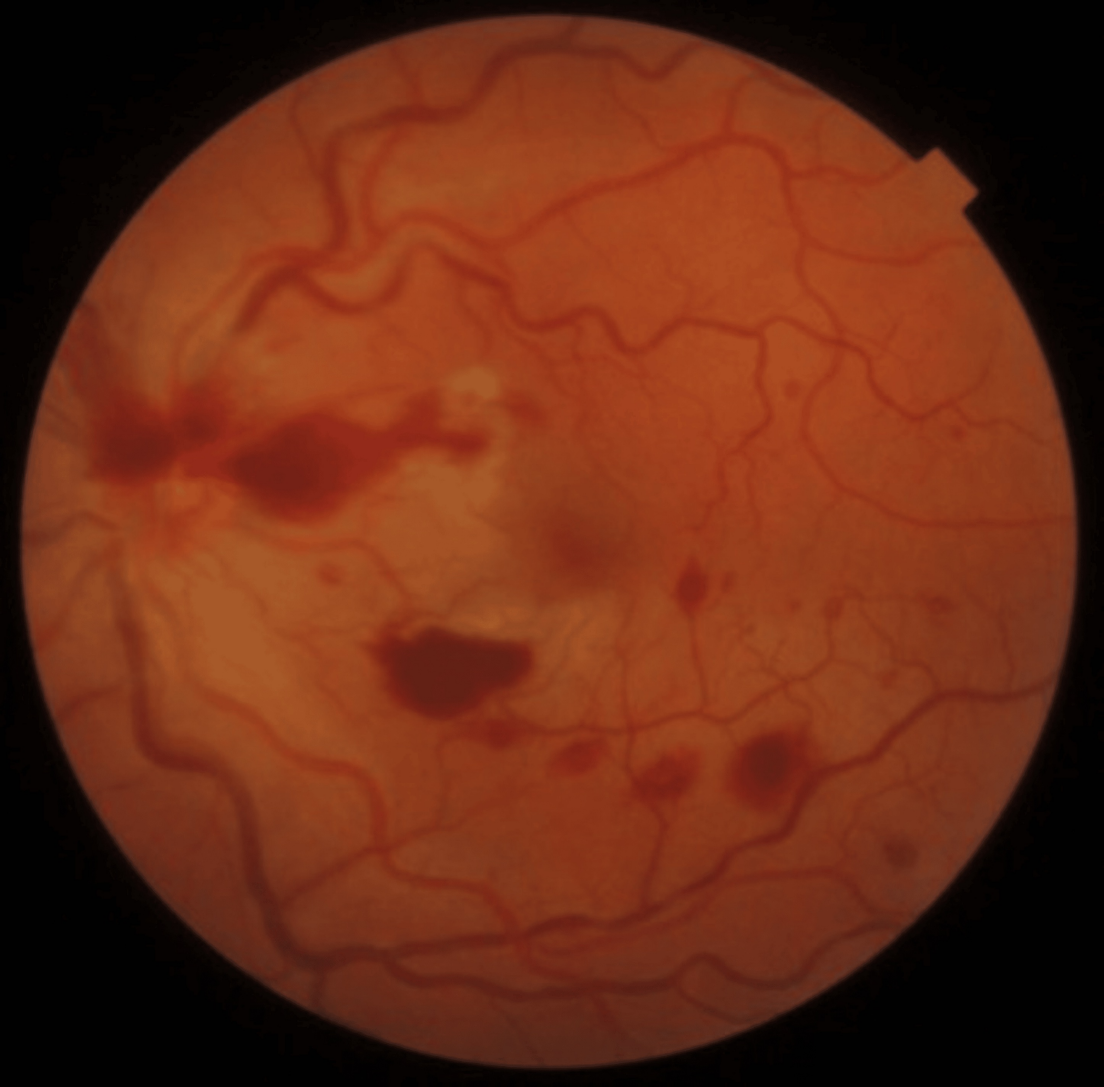
Left eye retinography at presentation. Whitish opacified retina in the papillomacular bundle, with dilated and tortuous retinal veins and intraretinal hemorrhages in the peripapillary region and along the inferotemporal vascular arcade. Image acquired on Topcon DRI Triton (Topcon, Tokyo, Japan).

**Figure 2:**
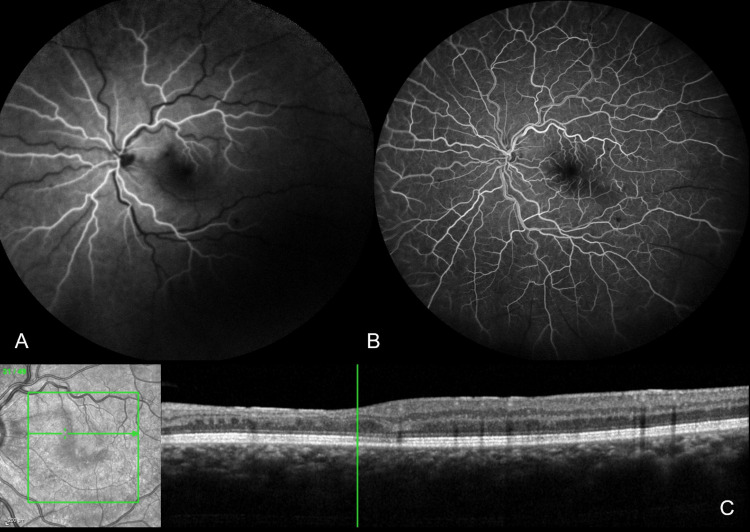
Imaging from the previous admission. Fluorescein angiography at 23″ (A) and 36″ (B) shows delayed venous filling. (C) SD-OCT revealed inner retinal thinning along the papillomacular bundle. Images acquired on Spectralis Heidelberg Retina Angiograph + OCT (Heidelberg Engineering, Heidelberg, Germany). SD-OCT: spectral-domain optical coherence tomography.

Visual acuity (VA) was 10/10 in the right eye and 8/10 in the left eye. Anterior segment examination was normal in both eyes, as was fundoscopy of the right eye. Fundoscopy of the left eye revealed a whitish opacified retina in the papillomacular bundle area, dilated tortuous retinal veins, and a few intraretinal hemorrhages (Figure 1). Review of the fluorescein angiogram performed during the previous admission revealed delayed venous filling, associated with vascular dilation and tortuosity, and a few scattered intraretinal hemorrhages (Figures 2A, 2B), consistent with early CRVO. Optical coherence tomography (OCT) showed inner retinal thinning in the papillomacular bundle area, with no macular edema (Figure 2C). These findings supported a revised diagnosis of CLRAO secondary to CRVO. A systemic work-up was repeated, this time focusing on venous system thrombotic risk factors, and revealed heterozygosity for the factor V Leiden mutation (Table 1). Hematology recommended prophylactic anticoagulation therapy before long-haul flights.

**Table 1:** Laboratory findings related to venous thrombophilia and vascular risk assessment. Laboratory evaluation showed normal coagulation, lipid, and glycemic profiles. Antiphospholipid antibody screening was negative. The only relevant abnormality was heterozygosity for the factor V Leiden mutation. PT: prothrombin time; aPTT: activated partial thromboplastin time; INR: international normalized ratio; LA: lupus anticoagulant; aCL: anticardiolipin antibodies; anti-β2GPI: anti-β2 glycoprotein I antibodies; HbA1c: glycosylated hemoglobin.

Category	Parameters	Result/interpretation
Hematology	Hemoglobin, platelets	Within normal limits
Coagulation profile	PT, INR, aPTT, fibrinogen	Normal
Natural anticoagulants	Antithrombin, protein C, protein S	Normal
Homocysteine	—	Normal
Genetic testing	Factor V Leiden	Heterozygous (abnormal)
	Prothrombin G20210A mutation	Not detected
Antiphospholipid antibodies	LA, aCL, anti-β2GPI	Negative
Metabolic profile	HbA1c, lipid panel	Normal

At the time of presentation, the patient was undergoing HOT, initiated shortly after the diagnosis of presumed isolated CLRAO. Despite ongoing treatment, he experienced a new acute episode, with fundoscopic findings suggestive of worsening venous stasis. Given the revised diagnosis of CLRAO associated with CRVO, and with the rationale of decreasing vascular permeability and decreasing hydrostatic pressure at the level of the capillary bed, it was decided to introduce intravitreal anti-VEGF therapy. The patient received two monthly intravitreal injections of bevacizumab (1.25 mg/0.05 mL), and HOT was continued concurrently for a further two weeks, following a standard protocol of 100% oxygen administered at 2.4 ATA (atmospheres absolute) for 90 minutes per session, completing a total of 12 sessions.

**Figure 3:**
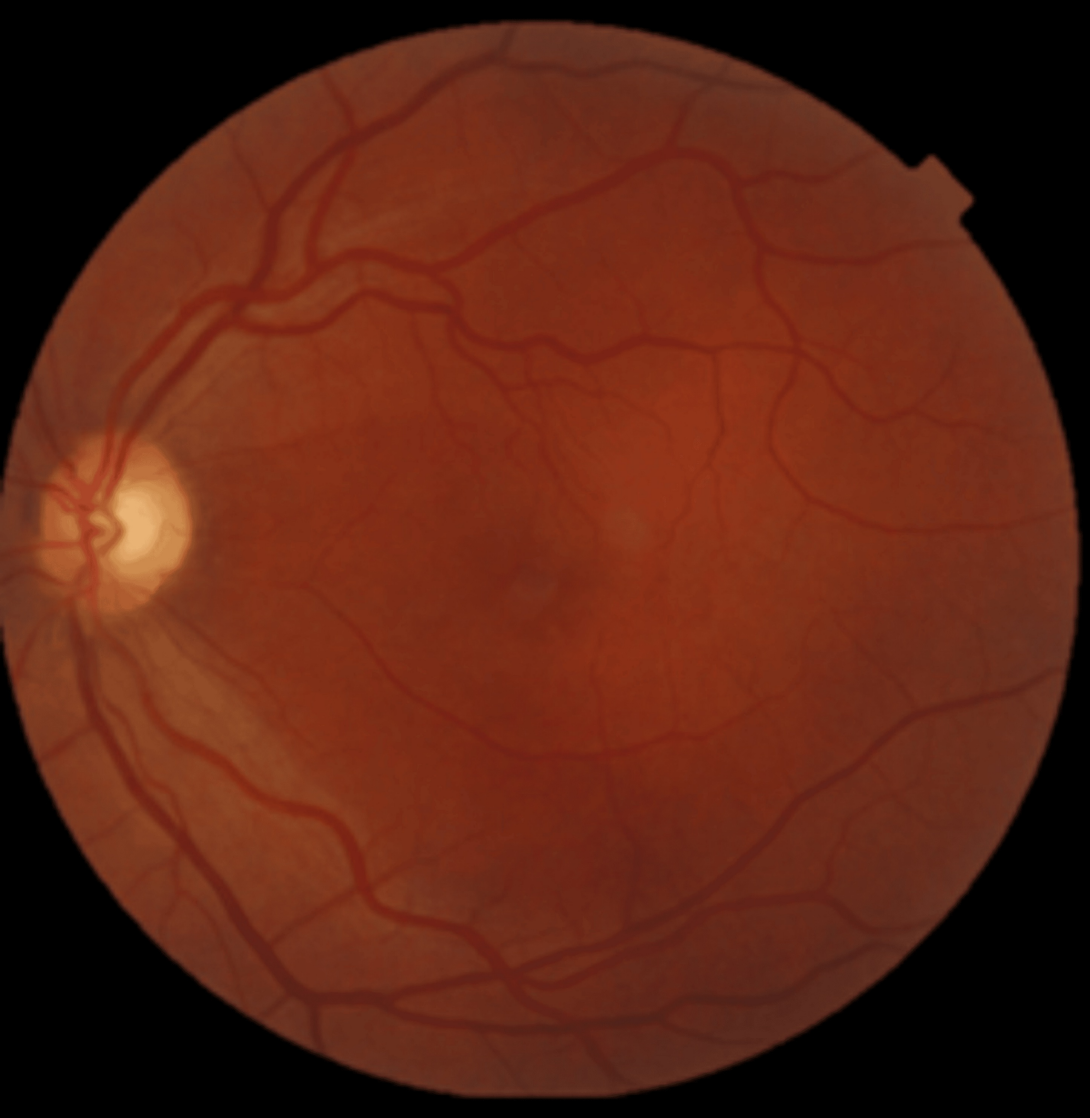
Fundus retinography at two-year follow-up. Complete resolution of venous stasis signs. Image acquired on Topcon DRI Triton (Topcon, Tokyo, Japan).

Following the combined treatment, the patient experienced complete visual recovery, with VA improving to 10/10 in the affected eye (OS). Fundoscopic signs of venous stasis progressively resolved, including normalization of venous caliber and disappearance of retinal hemorrhages (Figure 3). This favorable outcome was sustained over a follow-up period of two years, during which no recurrence of symptoms or structural complications was observed.

## Discussion

Combined CLRAO and CRVO account for approximately 40% of all CLRAO cases [7,8]. In a series by Hayreh et al., most eyes with this diagnosis presented in younger individuals, with a mean age of 45.8 years [2]. Notably, one-third of patients in this cohort reported transient visual blurring before persistent vision loss. Our patient also experienced two such episodes in the weeks preceding presentation.

The pathophysiology behind this association remains incompletely understood. The prevailing hypothesis is based on the distinct hemodynamic environment of the cilioretinal artery, which derives from the posterior ciliary circulation. Unlike the central retinal artery, this vascular territory lacks an autoregulatory mechanism and operates at a lower perfusion pressure [2,7]. In the event of CRVO, the rise in venous pressure reduces the arteriovenous perfusion gradient [4]. Additionally, thrombosis within a retinal vein leads to obstruction of blood flow, resulting in increased intraluminal pressure. According to Starling’s law, this promotes the transudation of plasma proteins and fluid into the retinal interstitium, thereby increasing interstitial oncotic pressure and perpetuating tissue oedema [5,8]. While the central retinal artery may compensate through autoregulation, the cilioretinal artery cannot, resulting in transient or sustained ischemia in the corresponding territory. This hemodynamic block occurs when either arterial inflow is reduced or venous outflow is elevated, effectively impairing cilioretinal perfusion [2,4].

It is worth noting that retinal capillary ischemia may also occur in varying and transient degrees due to increased intraluminal pressure within the capillary bed secondary to venous stasis. This has been described in paracentral acute middle maculopathy (PAMM), which results from hypoperfusion at the level of the deep capillary plexus, a watershed zone particularly susceptible to ischemic injury [9]. According to the Krogh cylinder model, such regions are especially vulnerable to hypoxia due to their limited perfusion reserve [10]. PAMM may manifest as transient scotomas and can precede more severe vascular events, reflecting early stages of the retinal ischemic cascade [11]. Interestingly, in a study by Pichi et al., PAMM was reported in all eyes with CLRAO, with or without CRVO, supporting the concept of cilioretinal artery insufficiency as a hypoperfusion phenomenon rather than a complete occlusion [12].

The initial diagnosis of isolated CLRAO likely represented a subclinical form of CRVO. The preceding episodes of transient visual disturbance support a diagnosis of evolving venous outflow obstruction with fluctuating hemodynamic compromise. Recognizing this association is clinically relevant, as it redirects the diagnostic work-up from arterial to venous thrombotic causes [13]. In our patient, this approach led to the identification of heterozygosity for the factor V Leiden mutation.

HOT is primarily used in retinal arterial occlusions based on the principle that the choroid is capable of supplying 100% of the oxygen needed by the inner and outer retinal layers under hyperoxic conditions [14,15]. In cases of CLRAO associated with CRVO, HOT may also provide hemodynamic benefits by reducing RVP through three complementary mechanisms: reduction of intraocular pressure, reduction of episcleral venous pressure, and a vasoconstrictive effect, which limits the transudation of blood components into the retinal interstitium [5]. Although evidence is lacking, a small number of published case reports have described favorable anatomical and functional outcomes with HOT in patients with this diagnosis [5,16,17]. In our case, HOT was initiated shortly after the initial diagnosis of CLRAO, but the patient developed worsening signs of venous stasis while still under treatment.

A review of the literature identified five published cases of CLRAO associated with CRVO treated with intravitreal anti-VEGF. Two cases were treated with a single injection of ranibizumab, two with a single injection of bevacizumab, and one with a loading dose regimen. All reported sustained resolution of venous stasis signs following treatment. However, in those where visual fields were assessed, a persistent scotoma remained in the distribution of the cilioretinal artery [3,6,18].

In retinal vein occlusions, VEGF is upregulated in the vitreous, neurosensory retina, and retinal pigment epithelium, leading to vascular hyperpermeability, disruption of the blood-retina barrier, and eventual neovascularization [19]. Intravitreal anti-VEGF therapy counters these effects by promoting vasoconstriction and reducing venous permeability, which may reduce the edema surrounding the occluded vein and improve perfusion in adjacent arterial territories [3,6]. Additionally, Kida et al. demonstrated that retinal RVP significantly decreases after a single intravitreal anti-VEGF injection in patients with RVO [20].

In our patient, a single injection of bevacizumab was administered after an incomplete response to HOT. The marked and sustained improvement in venous stasis observed suggests a potential synergistic effect, with HOT and anti-VEGF lowering RVP through different pathways.

Although causality cannot be established from a single case, the temporal association between treatment and the sustained anatomical and functional recovery makes the therapeutic hypothesis compelling. Nevertheless, this report’s single-case nature, absence of a control group, and lack of formal functional field testing limit causal inference and generalizability, and should be considered when interpreting the favorable outcome.

## Conclusions

To the best of our knowledge, this is the first reported case of CLRAO associated with CRVO treated with a combination of HOT and intravitreal anti-VEGF therapy. In combined CLRAO and CRVO, the rise in retinal venous pressure compromises cilioretinal artery perfusion, leading to inner retinal ischemia; addressing both hypoxia and venous hypertension is therefore pathophysiologically justified.

The favorable and sustained outcome observed supports the hypothesis that this combined approach may provide therapeutic benefit through complementary mechanisms: HOT enhances oxygen diffusion to the ischemic retina and reduces venous pressure, while anti-VEGF therapy decreases vascular permeability, induces vasoconstriction, and further lowers retinal venous pressure. This synergistic action may help restore hemodynamic balance and prevent irreversible ischemic damage in similar presentations. Registry-based and multicenter case-series documentation are needed to clarify which patients benefit, how treatment is delivered, and what outcomes are achieved with combined HOT and anti-VEGF therapy.
